# A VAM-Based Equivalent Model for Triangular Honeycomb Sandwich Panels: Comparison with Numerical and Experimental Data

**DOI:** 10.3390/ma15144766

**Published:** 2022-07-07

**Authors:** Zhen Wang, Xinlong Yang, Wengen Lai, Yifeng Zhong, Rong Liu

**Affiliations:** 1 School of Civil Engineering, Chongqing University, Chongqing 400045, China; 20194531@cqu.edu.cn (Z.W.); 20195013@cqu.edu.cn (X.Y.); 20154673@cqu.edu.cn (W.L.); 202016131332@cqu.edu.cn (R.L.); 2 Key Laboratory of New Technology for Construction of Cities in Mountain Area, Chongqing University, Chongqing 400045, China

**Keywords:** variational asymptotic method, triangular honeycomb sandwich panel, equivalent plate model, multiscale analysis, free and forced vibration

## Abstract

Due to their complex microstructures, the research on the static and dynamic behaviors of triangular honeycomb sandwich panels (triangular HSPs) is limited. In this study, the effective plate properties of triangular HSP was obtained by the homogenizing of the unit cell, and then the input to a VAM-based two-dimensional equivalent plate model (2D-EPM) to perform static and dynamic analyses. The accuracy of the proposed model for predicting the equivalent stiffness of the triangular HSP was verified by three-point bending experiments of 3D-printed specimens. Then, the static displacement, global buckling, and free vibrations predicted by 2D-EPM were verified with the results from three-dimensional finite element model simulations under various boundary conditions. The influences of structural parameters (including angle, core wall thickness, and cell side length of the unit cell) on the static and dynamic characteristics of triangular HSPs were also investigated, which can provide a useful tool for the modeling and evaluation of triangular HSPs under different conditions.

## 1. Introduction

The honeycomb sandwich structure originated in the field of bionics and was named after its resemblance to honeycombs. The honeycomb sandwich structure is a porous material with excellent characteristics, such as high strength, low weight, and thermal insulation. Honeycomb sandwich structures have been widely used in fields from aerospace to home decoration, and materials with this structure have attracted a considerable amount of research attention. Many theories, such as the Gibson formula [1,2], energy method [3], and homogenization method, have been developed to characterize the performances of honeycomb sandwich structures.

At present, a series of relevant theories, such as first-order shear theory (FOST) [4,5], high-order shear theory [6], layered theory [7], and zig-zag theory [8], have been proposed to examine the mechanical mechanism of the honeycomb sandwich panels. Huang et al. [9] developed a finite element model to investigate the vibration and damping of elastic–viscoelastic–elastic sandwich beams. To examine the dynamic characteristics of functionally graded porous sandwich plates, Gao et al. [10] developed a sandwich plate model by integrating the FOST, the equivalent theory of material mechanics, and the Newmark–Beta approach. Many studies have investigated sandwich panels by using high-order shear theory [11], with the displacement field serving as the high-order term of the thickness direction coordinates, to improve the calculation accuracy of the sandwich structure.

If the whole sandwich layer is modeled by first- or higher-order shear theory, it is collectively referred to as equivalent single-layer theory. However, a shear strain discontinuity exists in the equivalent single-layer theory at the layered interface of the structure. As a result, the delamination theory satisfying the interface continuity of the shear stress has also been developed [12]. Each layer has a corresponding displacement field, which can make the shear stress and displacement continuous, resulting in a good displacement response and stress distributions. However, there are certain disadvantages to the layered theory. In particular, the number of calculations of the model established according to the layered theory increases as the number of layers increases.

The dynamic characteristics of the honeycomb sandwich structure have also received a considerable amount of attention. There are many methods for analyzing the damping performance, including the complex eigenvalue technique (CET) [13] and the modal strain energy approach (MSE) [14]. Based on the MSE and the frequency variation characteristics of viscoelastic materials, Zhang et al. [15] studied the damping loss performances of frequency-varying-material composite sandwich structures through an iterative method. The main solution method for the issue of the frequency response is an iterative method, which combines the MSE and complex eigenvalue method to address the dynamic problems of the structure. Meunier et al. [16] determined the free vibrations and dynamic responses of composite sandwich panels by an iterative method based on the elastic viscoelastic correspondence principle. Moura [17] used an iterative technique to derive the damping and stiffness matrix based on the modal eigenvalues and eigenvectors and proposed a series of mass matrix processing methods to solve the dynamic problem of a frequency-varying viscoelastic interlayer.

The analytical theory of honeycomb sandwich panels began to be coupled with the finite element technique as a result of the aforementioned theoretical progress. For example, Farsani et al. [18] and Wu et al. [19] investigated the free vibrations of composite sandwich panels by using the FOST and global–local high-order deformation theory combined with the finite element method, respectively. As a result, numerical simulations have also become the main approach for investigating the mechanical properties of honeycomb sandwich panels, in addition to carrying out experiments to directly study such structures [20,21].

There are two main methods of numerical simulation for honeycomb sandwich structures. One is to directly establish a three-dimensional (3D) model of the honeycomb sandwich structure and carry out numerical simulation, but this approach is time consuming and labor intensive. The second is the micromechanical equivalent calculation of a honeycomb sandwich structure by using the Voight–Reuss formula [22], homogenization method [23,24], and other theories. Compared with the former, the latter saves more time and labor. The periodic unit cell is selected as the characterization unit, the equivalent properties are calculated, and the equivalent sandwich plate is taken as a homogeneous material. In this way, the challenges of complicated modeling and time-consuming calculations can be solved.

The “variational asymptotic method” (VAM) developed by Cesnik and Hodges has recently been extended to deal with periodic materials and structures [25,26,27]. The core of the VAM is to transform the difficulty of determining the definite solution of complex elasticity into a problem of asymptotically solving for the extreme value (or stationary value) of the functional, which is then summarized as solving the linear algebraic equations. The variational problem involved in mechanical problems is often associated with the energy principle. For example, if the system is in equilibrium, the stationary value of the system energy—the minimum potential energy principle—must be used. The original complicated 3D plate model can be made equivalent to a two-dimensional (2D) model by using the VAM, which has a high accuracy and has been applied in many areas [28,29,30,31].

At present, the research has mostly focused on hexagonal honeycomb sandwich panels, and their mechanical mechanisms are well understood. However, there has been less research on triangular honeycomb sandwich panels (triangular HSPs), and their static and dynamic behaviors remain unknown. One goal of our study is to reveal the mechanical mechanism of triangular HSPs by using a VAM-based equivalent plate model, as well as to determine the variation characteristics of its effective performance under various boundary conditions. Therefore, the starting point of this study focuses on the effective performance that is mainly concerned when the structure is applied in engineering, rather than the detailed study of physical properties.

The original problem of characterizing the effective performance of triangle HSPs has been solved in this work. Compared to the existing literature, the novelties of this work are that the effective plate properties of triangle HSPs were obtained by constitutive modeling over the unit cell, and inputted into the 2D-equivalent plate model (2D-EPM) for global analysis. The main contributions of the paper are the verification of the accuracy and validity of the present equivalent model in the global analysis of triangle HSPs, and the systematic analysis of the influence of parameters (including angle, core wall thickness, cell side length, and core form) on the effective performance (especially the specific stiffness) of triangle HSPs. To make the present work more self-contained, the authors have chosen to refer some text and equations from their previous publications.

## 2. Theoretical Foundation

### 2.1. 3D Energy Formulation of Triangular HSPs

**Figure 1:**
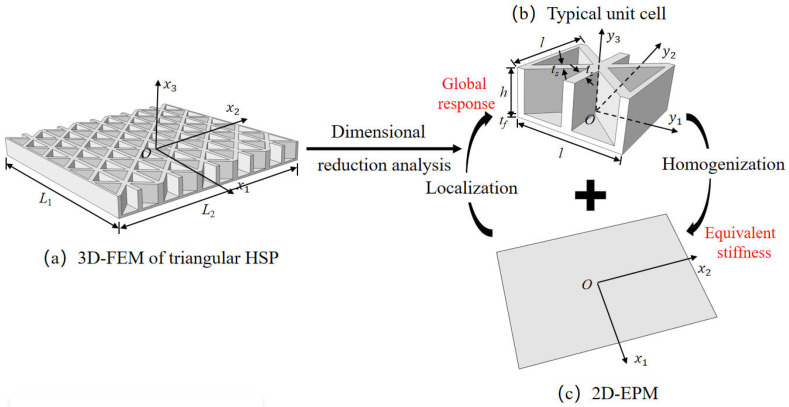
Analytic process of triangular HSPs by using the VAM-based equivalent model. (**a**) The 3D finite element model (3D-FEM), (**b**) constitutive modeling over the unit cell, and (**c**) 2D equivalent plate model (2D-EPM) (top facesheet removed for better view).

Figure 1 shows the analytic process of the triangular HSP by using a VAM-based equivalent model. From a macroscopic standpoint, the whole plate was equivalent to a continuous medium, with the displacement expected to vary slowly over one unit cell. From a microscopic standpoint, the displacement showed a considerable but small amplitude change over one unit cell. Hence, the displacements of the original triangular HSP may be represented by the displacements defined along the reference plane $x_{1}-x_{2}$ ($x_{3}$ disappears), with the partial derivative [31]

(1) $$\frac{\partial u\left(x_{\alpha };y_{i}\right)}{\partial x_{\alpha }}={\left.\frac{\partial u\left(x_{\alpha };y_{i}\right)}{\partial x_{\alpha }}\right|}_{y_{i}=const}+{\left.\frac{1}{\eta }\frac{\partial u\left(x_{\alpha };y_{i}\right)}{\partial y_{i}}\right|}_{x_{\alpha }=const}\equiv u_{,\alpha }+\frac{1}{\eta }u_{|i},$$

where $y_{i}\left(i=1,2,3\right)$ and $x_{\alpha }\left(\alpha =1,2\right)$ are micro- and macro-coordinates, and η is a small parameter indicating the scale ratio.

### 2.2. Step 1: Equivalent 2D Displacements from 3D Displacements

To develop the equivalent plate model of the triangular HSP by using the VAM, the 3D displacement field of the original triangular HSP $u_{i}$ must be represented by 2D plate variables ${\bar{u}}_{i}$, such that

(2) $$\begin{array}{cc} \; & u_{1}\left(x_{1},x_{2},y_{1},y_{2},y_{3}\right)=\underline{{\bar{u}}_{1}\left(x_{1},x_{2}\right)-\eta y_{3}{\bar{u}}_{3,1}}+w_{1}\left(x_{1},x_{2},y_{1},y_{2},y_{3}\right),\\ \; & u_{2}\left(x_{1},x_{2},y_{1},y_{2},y_{3}\right)=\underline{{\bar{u}}_{2}\left(x_{1},x_{2}\right)-\eta y_{3}{\bar{u}}_{3,2}}+w_{2}\left(x_{1},x_{2},y_{1},y_{2},y_{3}\right),\\ \; & u_{3}\left(x_{1},x_{2},y_{1},y_{2},y_{3}\right)=\underline{{\bar{u}}_{3}\left(x_{1},x_{2}\right)}+w_{3}\left(x_{1},x_{2},y_{1},y_{2},y_{3}\right), \end{array}$$

where ${\bar{u}}_{i}$ and $u_{i}$ are the displacements of the 2D-EPM and 3D-FEM, respectively, and $w_{i}$ represents unknown warping functions to be solved. The underlined terms may be interpreted as the deformation of the reference surface, which should meet the following constraints:

(3) $$\begin{array}{cc} \; & h{\bar{u}}_{a}\left(x_{\alpha }\right)=\langle u_{\alpha }\rangle +\langle \eta y_{3}\rangle {\bar{u}}_{3,2},\\ \; & h{\bar{u}}_{3}\left(x_{\alpha }\right)=\langle u_{3}\rangle , \end{array}$$

where 〈·〉 denotes the volume integral over the unit cell.

The 2D displacements are the averages of the 3D displacements if the origin of local coordinates was located at the geometric center of the unit cell, and the warping functions are constrained by

(4) $$\langle \eta w_{i}\rangle =0.$$

In the linear elastic stage, the 3D strain may be approximated by a linear form by using the decomposition of the rotation tensor [32], e.g.,

(5) $$\varepsilon_{ij}=\frac{1}{2}\left(\frac{\partial u_{i}}{\partial x_{j}}+\frac{\partial u_{j}}{\partial x_{i}}\right).$$

A variable change is implemented in the 3D warping functions,

(6) $$w_{i}\left(x_{1},x_{2},y_{3}\right)=\eta y_{3}\varphi_{i}\left(x_{1},x_{2}\right)+v_{i}\left(x_{1},x_{2},y_{3}\right),$$

where $\varphi_{1}$ and $\varphi_{2}$ represent the rotations of a transverse normal around the $x_{2}$ and $x_{1}$ axes, respectively, and $\varphi_{3}$ denotes the elongation of a transverse normal along the $x_{3}$ axis.

Substituting Equations (2) and (6) into Equation (5) and removing higher-order terms with negligible effect on the total energy yields explicit expressions of the 3D strain field:

(7) $$\begin{array}{cc} \; & \varepsilon_{11}=\epsilon_{11}+\eta y_{3}\kappa_{11}+\eta y_{3}\varphi_{1,1}+v_{1,1},\\ \; & 2\varepsilon_{12}=2\epsilon_{12}+2\eta y_{3}\kappa_{12}+\eta y_{3}\varphi_{2,1}+v_{2,1}+\eta y_{3}\varphi_{1,2}+v_{1,2},\\ \; & \varepsilon_{22}=\epsilon_{22}+\eta y_{3}\kappa_{22}+\eta y_{3}\varphi_{2,2}+v_{2,2},\\ \; & 2\varepsilon_{13}=\varphi_{1}+v_{1,3}+\eta y_{3}\varphi_{3,1}+v_{3,1},\\ \; & 2\varepsilon_{23}=\varphi_{2}+v_{2,3}+\eta y_{3}\varphi_{3,2}+v_{3,2},\\ \; & \varepsilon_{33}=\varphi_{3}+v_{3,3}, \end{array}$$

where $\epsilon_{\alpha \beta }$ and $\kappa_{\alpha \beta }$ can be defined as

(8) $$\epsilon_{\alpha \beta }\left(x_{1},x_{2}\right)=\frac{1}{2}\left({\bar{u}}_{\alpha ,\beta }+{\bar{u}}_{\beta ,\alpha }\right),\;\kappa_{\alpha \beta }\left(x_{1},x_{2}\right)=-{\bar{u}}_{3,\alpha \beta }.$$

The 3D strain field E may be represented as

(9) $$\begin{array}{c} E_{e}={ [\varepsilon_{11}\;\varepsilon_{22}\;2\varepsilon_{12}]}^{T}=\epsilon +\eta y_{3}\kappa +I_{\alpha }\left(\eta y_{3}\varphi_{∥,\alpha }+v_{∥,\alpha }\right),\\ 2E_{s}={ [2\varepsilon_{13}\;2\varepsilon_{23}]}^{T}=\varphi_{∥}+v_{∥,3}+e_{\alpha }\left(\eta y_{3}\varphi_{3,\alpha }+v_{3,\alpha }\right),\\ E_{t}=\varepsilon_{33}=\varphi_{3}+v_{3,3}, \end{array}$$

where the subscripts *e*, *s* and *t* denote in-plane, shear, and thickness, respectively; ${\left(\right)}_{\left|\right|}={ [{\left(\right)}_{1}\;{\left(\right)}_{2}]}^{T}$, $\epsilon ={ [\epsilon_{11}\;\;2\epsilon_{12}\;\;\epsilon_{22}]}^{T}$, $\kappa ={ [\kappa_{11}\;\kappa_{12}+\kappa_{21}\;\kappa_{22}]}^{T}$, and

(10) $$e_{1}=\left\{\begin{array}{c} 1\\ 0 \end{array}\right\},e_{2}=\left\{\begin{array}{c} 0\\ 1 \end{array}\right\},I_{1}= [\begin{array}{cc} 1 & 0\\ 0 & 1\\ 0 & 0 \end{array}],I_{2}= [\begin{array}{cc} 0 & 0\\ 1 & 0\\ 0 & 1 \end{array}].$$

### 2.3. Step 2: Energy Expression of the Triangular HSP

**Figure 2:**
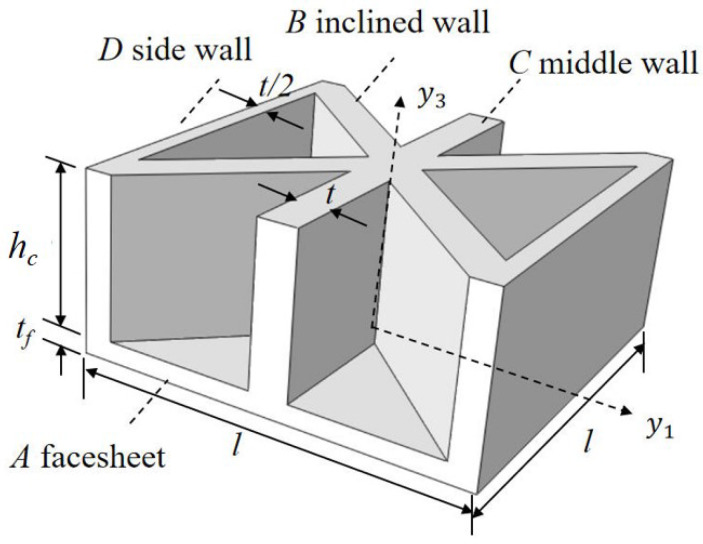
Integral domain division of unit cell (top facesheet removed for better view).

The panel’s strain energy may be expressed as

(11) $$U=\frac{1}{2}\int_{-b/2}^{b/2}\int_{-a/2}^{a/2}\frac{1}{\Omega }U_{\Omega }dx_{2}dx_{1},$$

where *b* and *a* are the width and length of the panel, respectively. $U_{\Omega }$ represents the strain energy over the domain of the unit cell, which can be expressed as

(12) $$\begin{array}{cc} 2U_{\Omega }= & 2\times \int_{-t_{f}}^{0}\int_{-\frac{l}{2}}^{\frac{l}{2}}\int_{-\frac{l}{2}}^{\frac{l}{2}}E_{A}^{T}D_{A}E_{A}dy_{1}dy_{2}dy_{3}\\ \; & +4\times \int_{0}^{h_{c}}\int_{-y_{1}+\frac{l}{2}-\frac{\sqrt{2}}{2}t_{}}^{-y_{1}-\frac{l}{2}+\frac{\sqrt{2}}{2}t_{}}\int_{\frac{t_{}}{2}}^{\frac{l-t_{}}{2}}E_{B}^{T}D_{B}E_{B}dy_{1}dy_{2}dy_{3}\\ \; & +4\times \int_{0}^{h_{c}}\int_{0}^{\frac{l}{2}}\int_{0}^{\frac{t_{}}{2}}E^{T}D_{C}E_{C}dy_{1}dy_{2}dy_{3}\\ \; & +4\times \int_{0}^{h_{c}}\int_{0}^{\frac{l}{2}}\int_{\frac{l-t_{}}{2}}^{2}E_{D}^{T}D_{D}E_{D}dy_{1}dy_{2}dy_{3}, \end{array}$$

where the subscripts *A*, *B*, *C*, and *D* represent the facesheet, inclined wall, middle wall, and side wall, respectively, as illustrated in Figure 2, $t_{f}$ and *t* are the thickness of the facesheet and core wall, respectively, $h_{c}$ is the height of the core layer, and *l* is the cell side length.

Equation (12) can be expressed compactly as

(13) $$U=\frac{1}{2}\langle E^{T}DE\rangle =\frac{1}{2}\langle {\left\{\begin{array}{c} E_{e}\\ 2E_{s}\\ E_{t} \end{array}\right\}}^{T} [\begin{array}{ccc} D_{e} & D_{es} & D_{et}\\ D_{es}^{T} & D_{s} & D_{st}\\ D_{et}^{T} & D_{st}^{T} & D_{t} \end{array}]\left\{\begin{array}{c} E_{e}\\ 2E_{s}\\ E_{t} \end{array}\right\}\rangle ,$$

where $D_{e},D_{es},D_{et},D_{s},D_{st}$, and $D_{t}$ are the corresponding sub-matrices of the 3D 6×6 material matrix.

The virtual work done by the external load may be expressed as

(14) $$\begin{array}{cc} W_{3D} & =W_{2D}+W^{*}\\ \; & =\int_{s}\left(p_{i}{\bar{u}}_{i}+q_{\alpha }\delta {\bar{u}}_{3,\alpha }\right)ds+\int_{s}\left(\langle f_{i}w_{i}\rangle +\tau_{i}h\varphi_{i}/2-\beta_{i}h\varphi_{i}/2\right)ds, \end{array}$$

where $\delta {\bar{W}}^{*}$ and $\delta {\bar{W}}_{2D}$ are the virtual work related to and independent of the warping function, respectively, *s* denotes the surface of the 2D-EPM, $-h\varphi_{i}/2$ and $h\varphi_{i}/2$ denote the warping displacements on the bottom and top surfaces, respectively, $f_{i}$ is the body force, $\beta_{i}$ and $\tau_{i}$ are the traction forces on the bottom and top surfaces, respectively, and the distributed forces and moments along the reference surface are defined as $p_{i}=\langle f_{i}\rangle +\tau_{i}+\beta_{i}$ and $q_{\alpha }=h/2\left(\beta_{\alpha }-\tau_{\alpha }\right)-\langle \eta y_{3}f_{\alpha }\rangle$, respectively.

The absolute velocity of a generic point in the triangular HSP may be expressed as

(15) $$v=V+\tilde{\omega }\left(\xi +w\right)+\dot{w},$$

where *V* is the absolute velocity of a point in the deformed reference surface, $\dot{w}=\partial w/\partial t$, $\tilde{\omega }$ denotes the inertial angular velocity, $\tilde{\omega }=-e_{ijk}\omega$, with $e_{ijk}$ as the permutation symbol, and $\xi ={\left[0\;\;0\;\;x_{3}\right]}^{T}$.

The kinetic energy of the panel may be represented as

(16) $$\begin{array}{cc} K & =\frac{1}{2}\int_{V}\rho v^{T}vdV=K_{2D}+K^{*}\\ \; & =\frac{1}{2}\int_{\Omega }\left(\bar{\rho }V^{T}V+2\omega^{T}\tilde{\rho \xi }V+\omega^{T}\Phi \omega \right)d\Omega \\ \; & +\frac{1}{2}\int_{V}\rho [{\left(\tilde{\omega }w+\dot{w}\right)}^{T}\left(\tilde{\omega }w+\dot{w}\right)+2{\left(V+\tilde{\omega }\xi \right)}^{T}\left(\tilde{\omega }w+\dot{w}\right)]dV, \end{array}$$

where ρ is the mass density, $\bar{\rho }=\langle \rho \rangle$, $\rho \xi ={\left\lfloor \begin{array}{ccc} 0 & 0 & \langle \eta y_{3}\rho \rangle \end{array}\right]}^{T}$, and $\Phi = [\begin{array}{ccc} \langle \eta y_{3}^{2}\rho \rangle & 0 & 0\\ 0 & \langle \eta y_{3}^{2}\rho \rangle & 0\\ 0 & 0 & 0 \end{array}]$.

The elastodynamic behavior of the triangular HSP is governed by the Hamilton principle

(17) $$\int_{t_{1}}^{t_{2}} [\delta \left(K_{2D}+K^{*}-U\right)+{\delta W}_{2D}+{\delta W}^{*}]dt=0,$$

where $t_{1}$ and $t_{2}$ are arbitrary fixed times.

A common way of solving Equation (17) is to assume a form of the unknown warping function $w_{i}$, so as to directly reduce the original 3D model to a 2D plate model. Such an assumption, however, may introduce inaccuracies into a triangular HSP made of high-porosity and periodic microstructures. The asymptotic analysis of the variational statement in Equation (17) may be employed to obtain the solution of $w_{i}$, which will be detailed next.

### 2.4. Step 3: Dimensional Reduction Analysis

To solve the unknown warping function $w_{i}$ based on the VAM, the order of each term in Equation (17) should first be assessed as

(18) $$\begin{array}{cc} \; & \epsilon_{\alpha \beta }∼h\kappa_{\alpha \beta }∼\varphi_{i}∼\xi ,\;v_{i}∼h\xi ,\;v_{∥;\alpha }∼w_{3;\alpha }∼\frac{h}{a}\xi ,\\ \; & v_{∥;3}∼v_{3;3}∼\xi ,\;hf_{\alpha }∼\tau_{\alpha }∼\beta_{\alpha }∼n\frac{h}{a}\xi ,\;hf_{3}∼\tau_{3}∼\beta_{3}∼n{\left(\frac{h}{a}\right)}^{2}, \end{array}$$

where *n* and ξ are the order of the material properties and the minimum strain, respectively.

According to the VAM, the terms of $K^{*}$ and ${\delta W}^{*}$ that are asymptotically smaller than other terms can be removed, and the the variational statement in Equation (17) can be rewritten as

(19) $$\int_{t_{1}}^{t_{2}} [\delta \left(K_{2D}-\int_{\Omega }U_{0}d\Omega \right)+{\delta W}_{2D}]dt=0,$$

where $U_{0}$ denotes the zeroth-order approximation strain energy, which can be obtained as

(20) $$2U_{0}=\langle \begin{array}{c} {\left(\epsilon +\eta y_{3}\kappa \right)}^{T}D_{e}\left(\epsilon +\eta y_{3}\kappa \right)+{\left(\varphi_{∥}+v_{∥,3}\right)}^{T}D_{s}\left(\varphi_{∥}+v_{∥,3}\right)\\ +2{\left(\epsilon +\eta y_{3}\kappa \right)}^{T}\left(D_{es}\left(\varphi_{∥}+v_{∥,3}\right)+D_{et}\left(\varphi_{3}+v_{3,3}\right)\right)\\ +2{\left(\varphi_{∥}+v_{∥,3}\right)}^{T}D_{st}\left(\varphi_{3}+v_{3,3}\right)+{\left(\varphi_{3}+v_{3,3}\right)}^{T}D_{t}\left(\varphi_{3}+v_{3,3}\right) \end{array}\rangle .$$

According to the principle of minimum potential energy, the warping function can be solved from

(21) $$\min_{\langle \eta w_{i}\rangle =0}U_{0}.$$

From Equation (20), the corresponding Euler–Lagrange equations are obtained as

(22) $$\begin{array}{cc} \; & { [{\left(\epsilon +\eta y_{3}\kappa \right)}^{T}D_{es}+{\left(\varphi_{∥}+v_{∥,3}\right)}^{T}D_{s}+\left(\varphi_{3}+v_{3,3}\right)D_{st}]}_{,3}=\lambda_{∥},\\ \; & { [{\left(\epsilon +\eta y_{3}\kappa \right)}^{T}D_{et}+{\left(\varphi_{∥}+v_{∥,3}\right)}^{T}D_{st}+\left(\varphi_{3}+v_{3,3}\right)D_{t}]}_{,3}=\lambda_{3}, \end{array}$$

where $\lambda_{\left|\right|}={ [\lambda_{1}\;\lambda_{2}]}^{T}$ and $\lambda_{3}$ are Lagrange multipliers.

The boundary conditions (BCs) of the bottom and top surfaces can be obtained as

(23) $$\begin{array}{cc} \; & { [{\left(\epsilon +\eta y_{3}\kappa \right)}^{T}D_{es}+{\left(\varphi_{∥}+v_{∥,3}\right)}^{T}D_{s}+\left(\varphi_{3}+v_{3,3}\right)D_{st}^{T}]}^{+/-}=0,\\ \; & { [{\left(\epsilon +\eta y_{3}\kappa \right)}^{T}D_{et}+{\left(\varphi_{∥}+v_{∥,3}\right)}^{T}D_{st}+\left(\varphi_{3}+v_{3,3}\right)D_{t}]}^{+/-}=0, \end{array}$$

where the superscript “+/−” denotes the terms at the top/bottom surface.

$v_{\left|\right|}$ and $v_{3}$ can be solved from these conditions as

(24) $$\begin{array}{cc} \; & v_{∥}={\langle -\left(\epsilon +\eta y_{3}\kappa \right){\bar{D}}_{es}{\left(D_{s}\right)}^{-1}\rangle }^{T},\\ \; & v_{3}=\langle -\left(\epsilon +\eta y_{3}\kappa \right){\bar{D}}_{et}{\left({\bar{D}}_{t}\right)}^{-1}\rangle , \end{array}$$

where

(25) $$\begin{array}{c} {\bar{D}}_{es}=D_{es}-{\bar{D}}_{et}{\left(D_{st}\right)}^{T}{\left({\bar{D}}_{t}\right)}^{-1},\\ {\bar{D}}_{et}=D_{et}-D_{es}{\left(D_{s}\right)}^{-1}D_{st},\\ {\bar{D}}_{t}=D_{t}-{\left(D_{st}\right)}^{T}{\left(D_{s}\right)}^{-1}D_{st}. \end{array}$$

Substituting Equation (24) in Equation (21), we obtain

(26) $$U_{2D}=\frac{1}{2}\langle {\left(\epsilon +\eta y_{3}\kappa \right)}^{T}K\left(\epsilon +\eta y_{3}\kappa \right)\rangle =\frac{1}{2}{\left\{\begin{array}{c} \epsilon \\ \kappa \end{array}\right\}}^{T} [\begin{array}{cc} A & B\\ B^{T} & D \end{array}]\left\{\begin{array}{c} \epsilon \\ \kappa \end{array}\right\},$$

where A,B, and D are the 3×3 tensile, tension-bending coupling, and bending stiffness matrices, respectively,

(27) $$\begin{array}{c} A=\langle K\rangle ,\;B=\langle \eta y_{3}K\rangle ,\;D=\langle \eta y_{3}^{2}K\rangle ,\\ K=D_{e}-{\bar{D}}_{es}D_{s}^{-1}D_{es}^{T}-{\bar{D}}_{et}D_{et}^{T}/{\bar{D}}_{t}. \end{array}$$

The constitutive relation could be obtained by defining the force resultants $N=\frac{\partial U_{2D}}{\partial \epsilon }$ and moment resultants $M=\frac{\partial U_{2D}}{\partial \kappa }$, as follows:

(28) $$\left\{\begin{array}{c} N_{11}\\ N_{22}\\ N_{12}\\ M_{11}\\ M_{22}\\ M_{12} \end{array}\right\}= [\begin{array}{cccccc} A_{11} & A_{12} & A_{16} & B_{11} & B_{12} & B_{16}\\ A_{12} & A_{22} & A_{26} & B_{12} & B_{22} & B_{26}\\ A_{16} & A_{26} & A_{66} & B_{16} & B_{26} & B_{66}\\ B_{11} & B_{12} & B_{16} & D_{11} & D_{12} & D_{16}\\ B_{12} & B_{22} & B_{26} & D_{12} & D_{22} & D_{26}\\ B_{16} & B_{26} & B_{66} & D_{16} & D_{26} & D_{66} \end{array}]\left\{\begin{array}{c} \epsilon_{11}\\ \epsilon_{22}\\ 2\epsilon_{12}\\ \kappa_{11}\\ \kappa_{22}\\ 2\kappa_{12} \end{array}\right\}.$$

Although Equation (28) is similar to the CLT based on the Kirchhoff-love assumption, the present model is asymptotically correct. The transverse normal and shear stresses can be shown to vanish, a direct result of model derivation rather than an a priori assumption. The macroscopic behavior of the panel is governed by the variational statement in Equation (19) that involves the 2D field variables. Hence, the 2D-EPM may be used to represent the original structure in the global displacement, buckling, and free vibration analyses by using a FE linear solver in the ABAQUS package.

## 3. Validation Example

**Figure 3:**
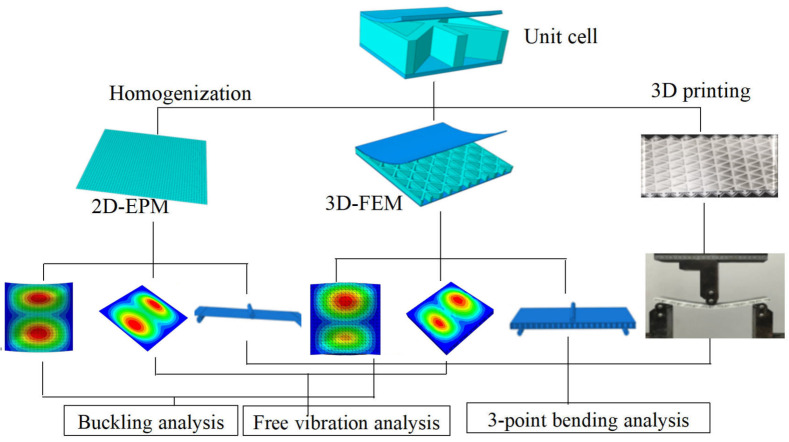
Comparison analysis process of the 3D-FEM, 2D-EPM, and experimental results.

In this section, numerical simulations and experiments are used to validate the accuracy of 2D-EPM. The comparison analysis process is shown in Figure 3. Compared with the bending test, the buckling and dynamic tests are more complex. This article mainly studies the stiffness, buckling eigenvalue and natural frequency of triangle HSPs, which are also mainly related to the equivalent stiffness. Therefore, the accuracy of different static and dynamic numerical simulation depends on the accuracy of calculated equivalent stiffness, which is verified by the bending test to a great extent.

The differences between the 3D-FEM, 2D-EPM, and experiment results are compared by using the following equations:

(29) $$Diff1=\frac{|2D-\mathrm{EPM}\;\mathrm{results}\;-\;\mathrm{Experimental}\;\mathrm{results}\;|}{\;\mathrm{Experimental}\;\mathrm{results}\;}\times 100\%,$$

(30) $$Diff2=\frac{|3D-\mathrm{FEM}\;\mathrm{results}\;-\;\mathrm{Experimental}\;\mathrm{results}\;|}{\;\mathrm{Experimental}\;\mathrm{results}\;}\times 100\%,$$

(31) $$Diff3=\frac{|2D-\mathrm{EPM}\;\mathrm{results}\;-\;3D-\mathrm{FEM}\;\mathrm{results}\;|}{\;3D-\mathrm{FEM}\;\mathrm{results}\;}\times 100\%.$$

The thicknesses of the top and bottom facesheets were 1 mm, and the thickness of the core layer was 8 mm. The core layer was created by repeating the core cell 13 times and 6 times along the $x_{1}$ and $x_{2}$ directions, respectively. The core cell was composed of two isosceles triangles and a partition wall, as shown in Figure 1b. The macroscopic dimensions of the panel were 260 mm × 130 mm, and the three baseline geometric parameters of the core cell used in the verification were the cell side length l=20 mm, core wall thickness t=2 mm, and included angle $\alpha =45^{\circ }$. The material properties of the test specimen and numerical model were the same: elastic modulus *E* = 2100 MPa, Poisson’s ratio μ=0.41, and density $\rho =1300\;\mathrm{kg}/m^{3}$. The indices C, S, and F represent clamped, simply supported, and free boundary conditions, respectively. The 3D-FEM and 2D-EPM respectively had 49,673 C3D10 and 4880 S4R elements after mesh convergence study. Table 1 shows the equivalent stiffness obtained by homogenizing the unit cell for reference.

### 3.1. Three-Point Bending Verification

Three-point bending experiments were performed by using a 50-kN screw-driven test machine (INSTRON 8832). The tests were carried out at a constant speed of 0.5 mm/min, with the applied load and central roller displacement recorded. A closed single-nozzle type was used in a 3D printer to ensure a higher accuracy of the printed specimens, and the printing material was a resin consumable with a diameter of 1.75 mm. All the 3D printing specimens had the same lengths and widths of 260 and 130 mm, respectively.

Table 2 compares the slopes of the displacement–load curves from the experiment and the 2D-EPM and 3D-FEM simulations under three-point bending. The values of Diff2 in the elastic stage were basically within 5.0%, indicating that the 3D-FEM can be used instead of experimental verification. Diff1 in the elastic stage was likewise less than 10%, indicating that it is reasonable to simulate the three-point elastic bending of the triangular HSP by using the 2D-EPM. The differences in experiments may come from the initial flaws of the samples. The printed 3D sample cannot reach the ideal homogeneity due to heating, nozzle pressure and environmental change. Furthermore, due to the limitation of the 3D printer, the top facesheet must be printed separately, and the top facesheet must be fixed on the printed core layer with glue of certain strength and adhesion, which will lead to differences in the connection strength between the top/bottom facesheet and the core layer. The difference in 3D-FEM and 2D-EPM may come from the boundary conditions utilised and the simplification of the model. The 2D-EPM greatly improves the calculation efficiency by removing high-order items in the calculation process, which would lead to the inevitable loss of accuracy.

**Figure 4:**
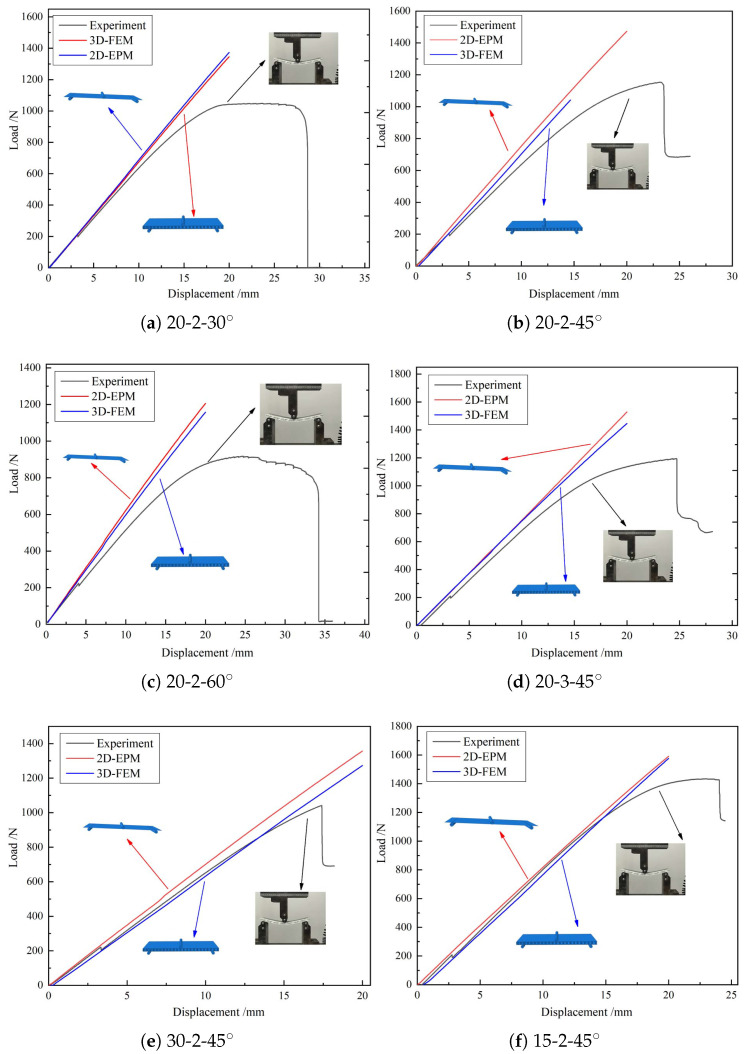
Displacement-load curves from experiments, 2D-EPM, and 3D-FEM (*l*-*t*-α denotes cell side length, core wall thickness and included angle).

The slopes corresponding to the displacement–load curves obtained from the three-point bending experiment and numerical simulation can be used to reflect the bending performances of the panels. Based on the principle of control variables, the triangular HSP with different core wall thicknesses *t*, including angles α, and cell side lengths *l* were selected to ensure the universality of the model verification. Figure 4 compares the displacement–load curves obtained from three-point bending tests, 3D-FEM and 2D-EPM simulations, and the images of the deformed test specimens are shown in the subfigure.

### 3.2. Global Buckling Verification

The 2D-EPM was used for buckling analysis to obtain the eigenvalues, and the findings are compared with the 3D-FEM results in this section. The boundary conditions (BCs) were simply supported on four sides (SSSS). A uniform load of 1/10 = 0.1 MPa and a line load of 1 N/mm were applied to the opposite sides of the 3D-FEM and 2D-EPM, respectively. The legends of the buckling modes are normalized for unified comparison.

Table 3 compares the first four buckling modes and loads predicted by different models. The maximum value of Diff3 was 6.68% in the first buckling mode, and the buckling modes predicted by the 3D-FEM and 2D-EPM were almost identical. That is, there were one, two, three, and four half-waves along the $x_{2}$ direction, whereas there was one half-wave along the $x_{1}$ direction in the first four buckling modes. Thus, it was proven that the 2D-EPM had high precision in analyzing the buckling behavior of triangle HSPs.

Table 4 compares the buckling modes and corresponding buckling loads predicted by different models under various boundary conditions. The errors of the buckling loads were within 5%, which would fully meet engineering requirements. The buckling modes predicted by the two models under various boundary conditions were consistent, illustrating the correctness of the 2D-EPM in buckling analysis under various boundary conditions.

### 3.3. Free Vibration Verification

Vibration modal analysis is the basis of structural dynamic response analysis. In this section, four boundary conditions (CCCC, CCCF, CCFF, and CFFF) were selected to examine the accuracy of the 2D-EPM in predicting the free vibrations. Table 5 compares the natural frequencies obtained by different models under the four boundary conditions. The natural frequencies increased as the boundaries became more constrained. The error of the natural frequencies was less than 6%, which would fully meet engineering requirements. The 2D-EPM can be used to replace the 3D-FEM to simulate the free vibrations of triangular HSPs.

To investigate the accuracy of the 2D-EPM for higher-order free vibration analysis, the first eight natural frequencies and vibration modes under CCCC boundary conditions are compared in Table 6. The legends of the vibration modes are normalized for unified comparison. It can be observed that the vibration modes predicted by different models were very consistent, with reasonably similar frequencies at different orders. The errors were within 6%, which would fully meet engineering requirements.

### 3.4. Comparison of Calculation Efficiencies

As shown in Table 7, compared with the 3D-FEM, the 2D-EPM has three advantages: (1) the definition of contact between the core layer and facesheet, the application of the load, and the boundary constraints are more convenient and concise; (2) different meshing of the 3D-FEM have a greater impact on the calculation speed and accuracy, whereas the meshing of 2D-EPM is faster and less difficult; and (3) the calculation efficiency of the 2D-EPM is nearly 50 times higher than that of 3D-FEM, for example, at 26 s with one CPU versus 9 min and 42 s with four CPUs in three-point bending analysis. The computer configuration included a Lenovo XiaoXinAir 15 ITL powered by an 11th Gen Intel i5-1135G7 CPU with a clock rate of 2.4 GHz and 16 GB of RAM.

In summary, using the 2D-EPM instead of the 3D-FEM to complete the numerical simulation would not only meet the engineering requirements but could also greatly improve the calculation efficiency and minimize the numerical simulation complexity. As a result, the following geometric study will use the 2D-EPM to investigate the influences of the geometric parameters on the static and dynamic behaviors of triangular HSPs.

## 4. Parametric Study

The effects of the core cell parameters (included angle, core wall thickness, and cell side length) on the equivalent stiffness, buckling load, and natural frequency of the triangular HSP are examined in this section. The macroscopic dimensions of the panel were 260 mm × 130 mm, and the BCs used for buckling and free vibration analysis were SSSS and CCCC, respectively.

### 4.1. Included Angle

**Figure 5:**
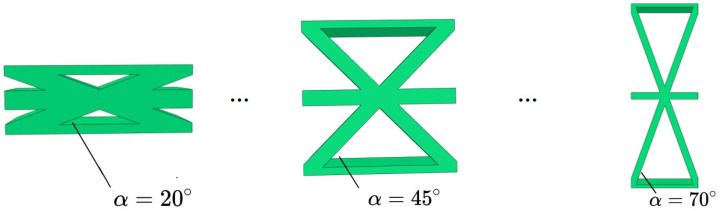
Geometric configurations of core cells with different included angles.

**Figure 6:**
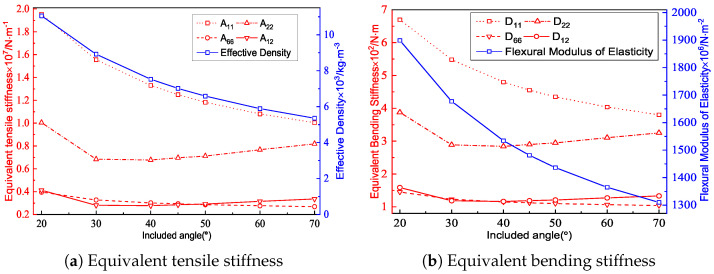
Effects of the included angles on the equivalent stiffness of the triangular HSP.

Figure 5 and Figure 6 show the geometric configurations of core cells with different included angles and their effects on the equivalent stiffness. There were three main factors affecting the equivalent tensile stiffness. The first was the filling degree of the core layer under the same macroscopic dimensions, which could be judged qualitatively based on the equivalent density. The second factor is the projected area of the core layer in different axial directions. The last factor is the in-plane equivalent elastic modulus.

The in-plane equivalent elastic modulus and the equivalent density decreased with an increasing included angle, resulting in a decrease in the equivalent tensile stiffness $A_{11}$. However, the tensile stiffnesses in the other directions decreased first and then increased because they were also affected by the change of the projected area in the corresponding axial direction. Thus, the equivalent plate showed distinct anisotropy. As shown in Figure 6b, the variation trend of the equivalent bending stiffness was similar to that of the tensile stiffness, mainly because the bending-resistance ability of the panel was mainly provided by the normal stress in the facesheets and the core far from the central layer. Furthermore, the flexural equivalent elastic modulus affected the bending stiffness in a proportional manner. As the flexural equivalent elastic modulus decreased with increasing included angle, so did the equivalent bending stiffness. As a result, from an economic standpoint, choosing a panel with a smaller included angle is not always the best option.

**Figure 7:**
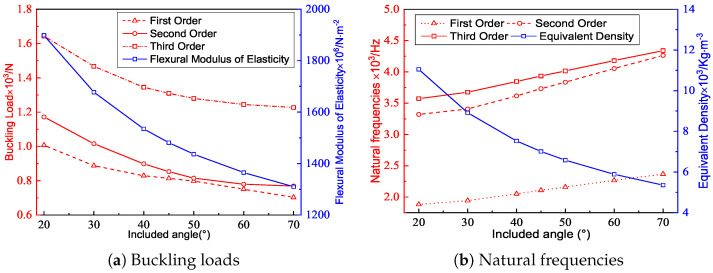
Effects of the included angles on the buckling loads and natural frequencies of the triangular HSP.

Figure 7 shows the effect of the included angle on the buckling load and natural frequency under the same conditions. The equivalent density of the triangular HSP decreased with an increasing included angle, indicating that the filling degree of the core layer diminished. The buckling load was related to the Poisson’s ratio, BCs, and elastic modulus at the macroscopic level. The equivalent elastic modulus of the triangular HSP decreased with increasing included angle, so the corresponding anti-buckling capacity also decreased. From a microscopic perspective, the calculated length of the facesheet without core support increased with increasing included angle, resulting in a decrease in the buckling load.

Figure 7b shows that the natural frequency of the triangular HSP increased with the increasing included angle. The natural frequency was positive proportional to the equivalent stiffness, while also inversely proportional to the equivalent density. The equivalent density decreased and the equivalent stiffnesses $A_{11}$ and $D_{11}$ increased with the increasing included angle. As a result of the combined impact of the two factors, the natural frequency increased.

### 4.2. Core Wall Thickness

**Figure 8:**
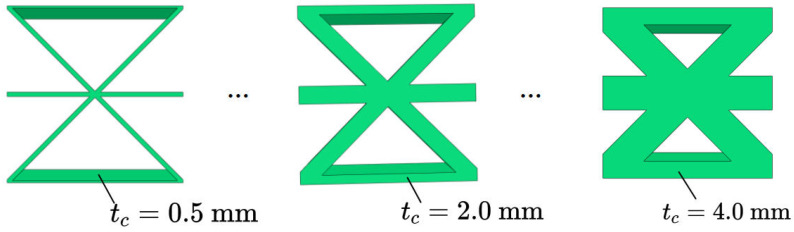
Geometric configurations of core cells with different core wall thicknesses.

**Figure 9:**
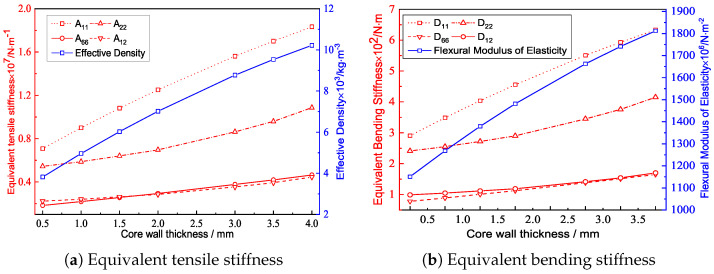
Effects of the core wall thicknessews on the equivalent stiffness of the triangular HSP.

Figure 8 and Figure 9 show the the geometric configurations of core cells with different core wall thicknesses and their effects on the equivalent stiffness. The equivalent elastic modulus and equivalent density increased with increasing core wall thickness. That is, the filling degree of the core layer and the contact area between the core layer and the facesheet increased with the same macroscopic dimensions, resulting in an increase in tensile stiffness. Furthermore, the change of each stiffness component was not the same when the core wall thickness increased by the same amount.

Figure 9b shows that the variation trend of the equivalent bending stiffness was similar to that of equivalent tensile stiffness, and both increased with increasing core wall thickness. The reason may be that the bending capacity of the plate mainly determined by the part away from the neutral axis of the plate. The sectional moment of inertia of the plate increased with increasing core wall thickness, so that the moment of the bending equivalent elastic modulus also increased with increasing core wall thickness.

**Figure 10:**
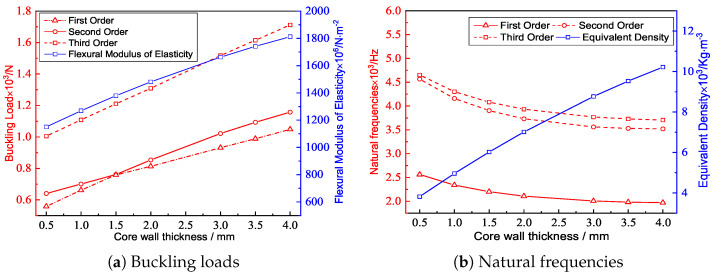
Effects of the core wall thicknesses on the buckling loads and natural frequencies of the triangular HSP.

Figure 10 shows the influence of the core wall thickness *t* on the buckling load and natural frequency of the triangular HSP. From a macroscopic standpoint, the equivalent elastic modulus of the 2D-EPM increased with the increase of core wall thickness, so the buckling load of the panel also increased. From a microscopic standpoint, increasing the core wall thickness reduced the calculated length of the panel, which would improve the buckling resistance of the panel. The effect of increasing the equivalent density caused by the increasing core wall thickness on the natural frequency was greater than that of increasing the equivalent stiffness, so the natural frequency of the triangular HSP decreased. In practice, a high anti-buckling capacity can be realized by increasing the core wall thickness and the boundary constraints, whereas a higher frequency can be obtained by reducing the core wall thickness and increasing the boundary constraints.

### 4.3. Cell Side Length

**Figure 11:**
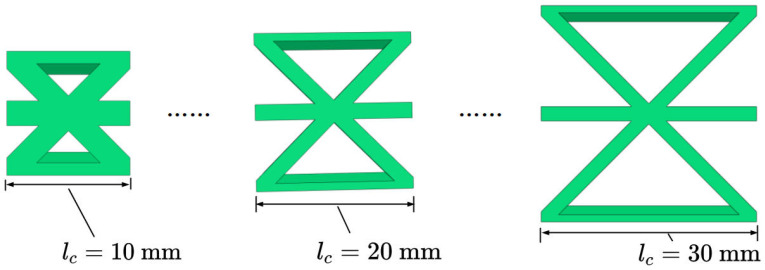
Geometric configuration of core cells with different cell side lengths.

**Figure 12:**
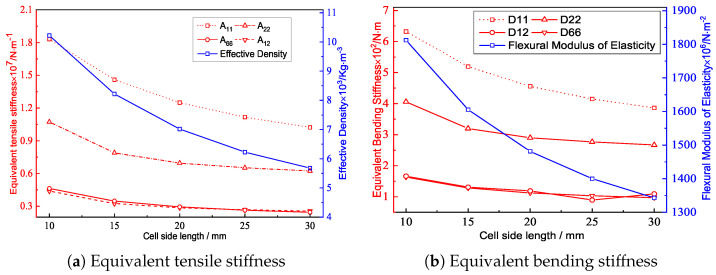
Effects of the cell side lengths on the equivalent stiffness of the triangular HSP.

Figure 11 and Figure 12 show the the geometric configurations of core cells with different cell side lengths and their effects on the equivalent stiffness. Figure 12a shows that the equivalent tensile stiffness decreased gradually as the cell side length increased. The main reason was that increasing the cell side length reduced the equivalent density and in-plane equivalent elastic modulus. Figure 12b shows that the equivalent bending stiffness decreased gradually with increasing cell side length, which was consistent with the change of the equivalent tensile stiffness.

**Figure 13:**
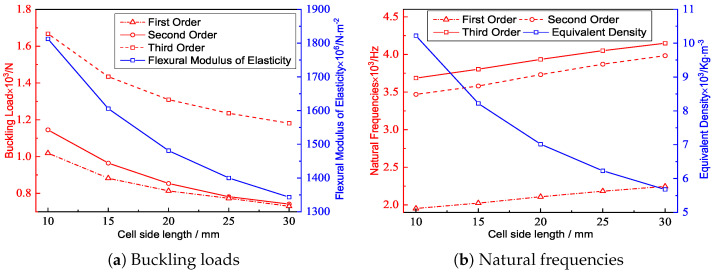
Effects of the cell side lengths on the buckling loads and natural frequencies of the triangular HSP.

Figure 13 shows the effect of the cell side length on the buckling load and natural frequency of the triangular HSP. From a macroscopic standpoint, the equivalent elastic modulus of the 2D-EPM decreased with increasing cell side length, so the buckling load also decreased. From a microscopic standpoint, increasing the cell side length would increase the calculated length of the panel and further reduce the buckling load. The effect of the decreasing equivalent density caused by the increase in the cell side length on the natural frequency was greater than that of the decreasing equivalent stiffness, so the natural frequency of the triangular HSP increased. A high anti-buckling capacity of the triangular HSP can be realized by decreasing the cell side length.

### 4.4. Specific Stiffness

**Figure 14:**
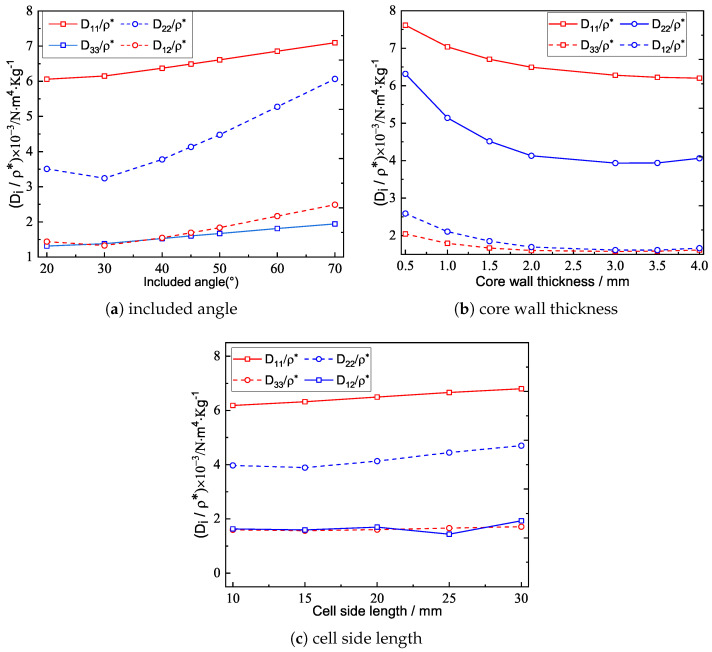
Specific stiffnesses of the triangular HSP corresponding to different parameters.

Figure 14a shows the specific stiffness (equivalent stiffness-to-density ratio) of the triangular HSP corresponding to different included angles. The specific stiffnesses mainly increased with the increasing included angle, indicating that a panel with a larger included angle could improve the bending performance. Therefore, it is necessary to choose the in-plane core layout with a larger included angle to make full use of the bending performance in practical use.

Figure 14b shows the specific stiffness of the triangular HSP corresponding to different core wall thicknesses. The specific stiffness decreased as the core wall thickness increased, and the reduction rate decreased as well. In practice, if there were no additional special requirements or restrictions on the core wall thickness, the triangular HSP with thinner core wall thickness should be used since it can improve the bending performance of the panel.

Figure 14c shows the specific stiffness of the triangular HSP corresponding to different cell side lengths. It can be found that with the increase of the cell side length, the specific stiffness of $D_{11}/\rho^{*}$ and $D_{22}/\rho^{*}$ increased, while the changes of $D_{33}/\rho^{*}$ and $D_{12}/\rho^{*}$ were not monotonic. In addition, the change range of the specific stiffness was not obvious with the increase of the cell side length. Therefore, the effect of cell side length on the specific stiffness should be placed secondarily in practical application.

## 5. Comparison of Different Core Forms

In addition to the triangular HSP, there are many other forms of honeycomb cores, such as diamond, orthogrid, and X-shaped, as illustrated in Table 8. To investigate the influence of the core forms on the static and dynamic behaviors of the panel based on the principle of control variables, the sandwich plates only had different core forms, and the cell and macroscopic dimensions were the same. The geometric parameters and other material properties were the same as those in Section 3.

### 5.1. Bending Performance

**Figure 15:**
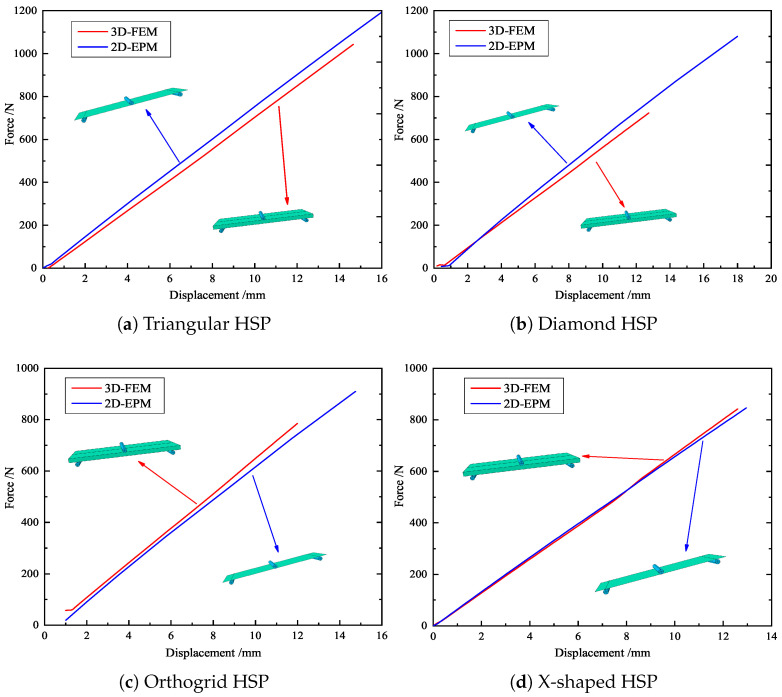
Comparison of displacement–load curves of HSPs with different core forms predicted by different models.

Three-point bending numerical simulations of four HSPs with different core forms were carried out by using different models. On this basis, the bending capacities of the four sandwich panels were compared according to the slopes of displacement–load curves. Figure 15 shows the simulation results of the 2D-EPM and 3D-FEM. The errors between the simulation results of the different models were small, and the two curves were approximately coincident, indicating that the numerical simulation results were relatively reliable and that these simulations can be used for detailed analysis. Furthermore, the triangular HSP exhibited the strongest bending performance (slope = 72 N/mm), followed by X-shaped HSP (slope = 65 N/mm), diamond HSP (slope = 64 N/mm), and finally, the orthogrid HSP (slope = 58 N/mm), which was mainly related to the sectional bending modulus of the $x_{1}$–$x_{3}$ plane.

### 5.2. Global Buckling

The global buckling behaviors of four sandwich panels with the same dimensions were numerically simulated by using the 2D-EPM and 3D-FEM under SSSS boundary conditions and bi-axial loading. A plate with dimensions of 240 mm × 240 mm was selected for buckling analysis. The dimensions were different from those of the previous buckling example in Section 3 to verify that the simulation results were independent of the panel size after the convergence study.

Table 9, Table 10, Table 11 and Table 12 compare the buckling modes and loads of the diamond HSP, orthogrid HSP, X-shaped HSP, and resized triangular HSP, respectively, and the relative errors of the buckling loads were indicated by red numbers in brackets. The first four buckling loads predicted by the different models were essentially the same, and the errors were within 10%, which fully meets engineering requirements. The first- and fourth-order buckling modes of the two models were basically the same, which were the f(1,1) and f(2,2) modes, respectively (f(m,n) denotes the mode shape with *m* and *n* representing the number of half-waves along the $x_{1}$ and $x_{2}$ directions, respectively), whereas the second- and third-order buckling modes were slightly different, which may have been due to the different equivalent stiffnesses along the $x_{1}$ and $x_{2}$ axes caused by different core forms.

The first four buckling loads of the four HSPs were not significantly different, but they showed different anti-buckling abilities. The first buckling load of the X-shaped HSP was the largest, followed by that of the triangular HSP, and that of the orthogrid HSP was the smallest. The variation of the second buckling load was still similar than that of the first buckling load, but the third buckling load of the triangular HSP was significantly larger. This was related to the sectional moments of inertia, which were in the order of X-shaped HSP > triangular HSP > diamond HSP > orthogrid HSP, and the buckling loads were also arranged in this order.

### 5.3. Free Vibrations

Table 13, Table 14, Table 15 and Table 16 compare the first four free vibrations of four HSPs with the same dimensions predicted by different models, and the red numbers in brackets indicated the relative errors of the natural frequencies. The vibration modes predicted by the two models were almost identical, and the differences of the natural frequencies between the two models were less than 10%, which would meet engineering requirements. The natural frequencies of the triangular HSP were the smallest, and those of the triangular HSP were the largest. The reason was mainly related to the equivalent elastic moduli and equivalent densities of the HSPs with different core forms.

## 6. Conclusions

Based on the VAM, the equivalent plate model was developed to investigate the global behavior of triangular HSPs. The following conclusions can be drawn from analyzing the influence of structural parameters on the static and dynamic characteristics of triangular HSPs.

(1) The 2D-EPM of triangular HSPs has high accuracy and efficiency. In the three-point bending simulation, the maximum slope error of displacement-load curve between 2D-EPM and experimental result is 6.60% and the minimum is 1.33%. In the buckling analysis, the maximum error of buckling load between 3D-FEM and 2D-EPM is 6.68% and the minimum error is 0.06%. In free vibration, the maximum error of natural frequencies between 2D-EPM and 3D-FEM is 3.67%, and the minimum error is 0.53%. The above errors are less than 10%, indicating that using 2D-EPM instead of 3D-FEM meets the engineering requirements. Moreover, the calculation efficiency of 2D-EPM is more than 50 times that of 3D-FEM, and 2D-EPM is better than 3D-FEM in contact definition between the core layer and facesheets, as well as application of load and the boundary constraint.

(2) The changes of the included angle α, cell side length $l_{c}$ and core wall thickness *t* would affect the effective plate properties. The equivalent stiffness and buckling load decrease as well as the natural frequency increases with increasing included angle, decreasing core wall thickness and increasing cell side length. Compared with other three HSPs with different core forms, the triangular HSP not only has excellent bending resistance, but also has better buckling resistance. The research focus on the free vibration of triangular HSP, and the impact resistance of triangular HSP can be further investigated on this basis.

## Figures and Tables

**Table 1:** Equivalent stiffness of the triangle HSP (unit: SI).

1.25 × 10${}^{4}$	2.85 × 10${}^{3}$	7.31 × 10${}^{-2}$	6.25 × 10${}^{4}$	1.43 × 10${}^{4}$	3.28 × 10${}^{-2}$
	6.96 × 10${}^{3}$	1.79 × 10${}^{-1}$	1.43 × 10${}^{4}$	3.48 × 10${}^{4}$	8.15 × 10${}^{-2}$
		2.94 × 10${}^{3}$	−1.88 × 10${}^{-1}$	−4.59 × 10${}^{-1}$	1.47 × 10${}^{4}$
	sym.		4.55 × 10${}^{5}$	1.19 × 10${}^{5}$	−2.40 × 10${}^{0}$
				2.90 × 10${}^{5}$	−5.86 × 10${}^{0}$
					1.12 × 10${}^{5}$

**Table 2:** Comparison of the slopes of displacement–load curves from the experiments, 2D-EPM, and 3D-FEM with different structural parameters under three-point bending.

Type of Panel *l*-*t*-α	2D-EPM N/mm	3D-FEM N/mm	Test Results N/mm	Diff2	Diff1
20-2-30${}^{\circ }$	69.24	67.88	66.33	2.34%	4.39%
20-2-45${}^{\circ }$	72.22	70.05	67	4.55%	7.79%
20-2-60${}^{\circ }$	59.08	56.09	53.95	3.97%	9.51%
20-3-45${}^{\circ }$	76.67	72.85	70.07	3.97%	9.42%
30-2-45${}^{\circ }$	68.33	64.1	65.46	2.08%	4.38%
15-2-45${}^{\circ }$	81.4	80.33	84.35	4.77%	3.50%

**Table 3:** Comparison of the buckling modes and buckling loads of the triangular HSP under simply supported boundary conditions predicted by different models.

Model	1	2	3	4
3D-FEM	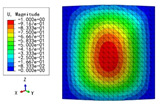	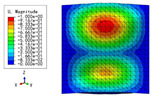	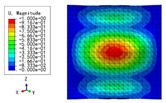	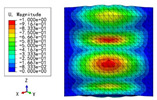
	1123.4 N	1126.8 N	1661.1 N	2235.3 N
2D-EPM	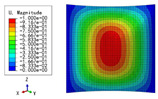	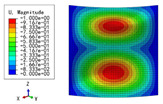	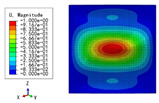	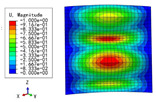
	1048.0 N	1158.0 N	1711.7 N	2347.9 N
Diff3	6.68%	2.84%	3.01%	5.01%

**Table 4:** Comparison of the buckling modes and loads of the triangular HSP under various boundary conditions predicted by different models.

Items	CSFS	CSFC	FSFC	CSCC
B.C.	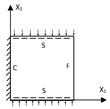	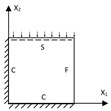	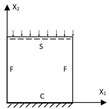	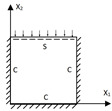
3D-FEM	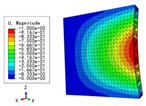	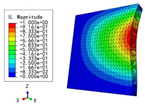	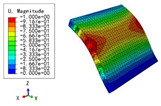	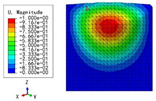
	241.13 N	416.12 N	273.03 N	1222.9 N
2D-EPM	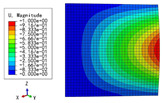	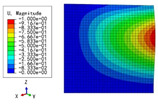	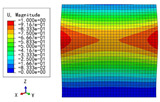	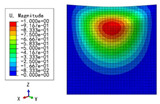
	238.44 N	416.37 N	271.25 N	1265.7 N
Diff3	1.12%	0.06%	0.65%	3.50%

**Table 5:** Comparison of the first four natural frequencies (Hz) of the triangle HSP predicted by different models.

Orders	CCCC			CCCF		
	3D-FEM	2D-EPM	Diff3	3D-FEM	2D-EPM	Diff3
1	1965.0	1977.0	0.61%	1364.0	1420.8	4.16%
2	3553.4	3534.4	0.53%	2117.1	2077.8	1.86%
3	3769.1	3712.3	1.51%	3301.6	3473.8	5.22%
4	5062.7	4919.2	2.83%	3727.2	3569.5	4.23%
**Orders**	**CCFF**			**CFFF**		
	**3D-FEM**	**2D-EPM**	**Diff3**	**3D-FEM**	**2D-EPM**	**Diff3**
1	402.46	401.77	0.17%	218.43	221.75	1.52%
2	1299.4	1233.1	5.10%	480.15	472.98	1.49%
3	1505.9	1458.9	3.12%	1186.5	1172.8	1.15%
4	2460.4	2314.0	5.95%	1235.0	1236.0	0.08%

**Table 6:** Comparison of the higher-order vibration modes and the corresponding frequencies under CCCC boundary conditions predicted by different models.

Orders	1	2	3	4
3D-FEM	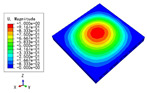	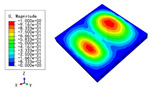	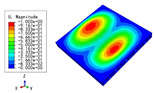	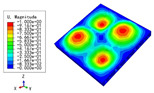
	1965.0 Hz	3553.4 Hz	3769.1 Hz	3769.1 Hz
2D-EPM	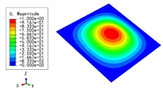	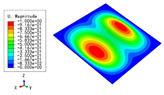	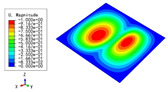	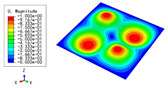
	1977.0 Hz	3534.4 Hz	3712.3 Hz	3712.3 Hz
Diff3	0.61%	0.53%	1.51%	2.83%
**Orders**	**5**	**6**	**7**	**8**
3D-FEM	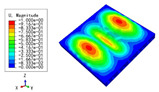	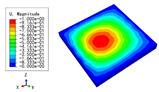	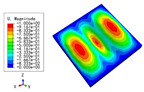	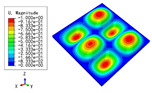
	5062.7 Hz	5673.3 Hz	6084.8 Hz	6441.5 Hz
2D-EPM	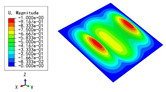	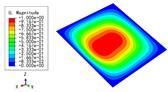	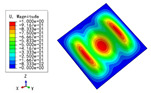	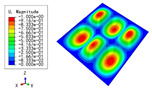
	4919.2 Hz	5581.3 Hz	6280.8 Hz	6821.7 Hz
Diff3	1.62%	3.67%	3.22%	5.90%

**Table 7:** Comparison of the calculation efficiencies between the different models.

Items		3D-FEM	2D-EPM	
			Unit Cell	2D Plate
Element type		C3D10	C3D10	S4R
Number of elements		49,673	30,907	4880
Number of nodes		82,937	49,116	5485
Global	3-point bending	113 min	–	128 s
response	Buckling	55 min	–	52 s
analysis	Vibration	24 min	–	48 s

**Table 8:** Geometries of honeycomb sandwich panels with different core forms.

Items	Triangular HSP	Diamond HSP	Orthogrid HSP	X-Shaped HSP
Core forms	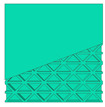	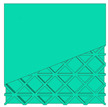	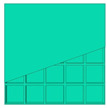	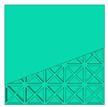
Core cell	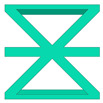	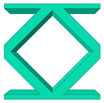	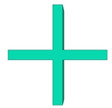	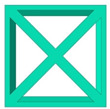

**Table 9:** Comparison of the buckling modes and buckling loads of the diamond HSP under SSSS boundary conditions predicted by different models.

Model	1	2	3	4
3D-FEM	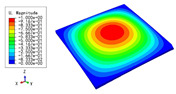	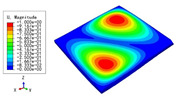	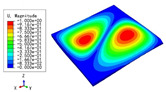	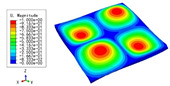
	59.164 N	143.93 N	144.00 N	233.46 N
2D-EPM	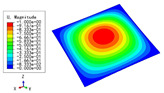	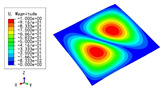	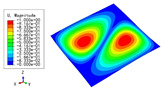	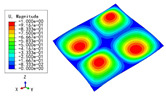
	55.210 N (6.68%)	139.60 N (3.01%)	139.60 N (3.06%)	230.84 N (1.12%)

**Table 10:** Comparison of the buckling modes and buckling loads of the orthogrid HSP under SSSS boundary conditions predicted by different models.

Model	1	2	3	4
3D-FEM	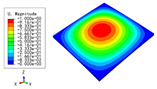	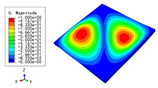	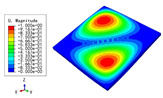	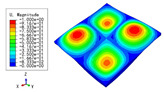
	49.979 N	123.21 N	125.75 N	213.54 N
2D-EPM	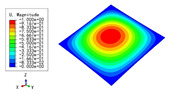	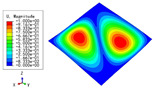	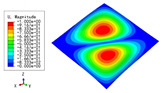	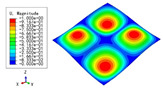
	52.918 N (5.88%)	134.21 N (8.93%)	134.21 N (6.73%)	221.40 N (3.68%)

**Table 11:** Comparison of the buckling modes and buckling loads of the X-shaped HSP under SSSS boundary conditions predicted by different models.

Model	1	2	3	4
3D-FEM	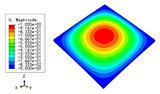	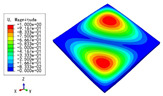	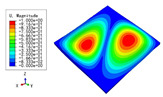	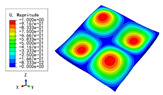
	59.164 N	143.93 N	145.39 N	238.09 N
2D-EPM	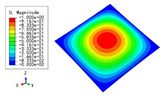	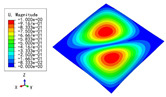	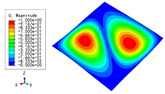	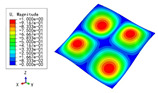
	60.288 N (1.90%)	153.19 N (6.43%)	153.19 N (5.36%)	253.67 N (6.54%)

**Table 12:** Comparison of the buckling modes and buckling loads of the resized triangular HSP under SSSS boundary conditions predicted by different models.

Model	1	2	3	4
3D-FEM	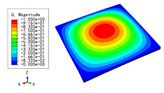	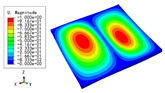	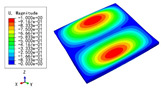	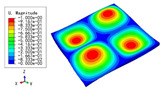
	58.933 N	138.83 N	148.01 N	232.60 N
2D-EPM	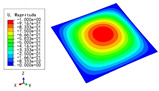	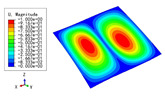	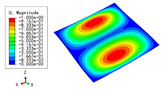	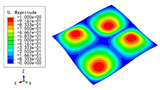
	56.867 N (3.51%)	135.33 N (2.52%)	155.60 N (5.13%)	240.27 N (3.30%)

**Table 13:** Comparison of the first four free vibrations of the diamond HSP under CCCC boundary conditions predicted by different models.

Model	1	2	3	4
3D-FEM	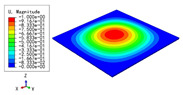	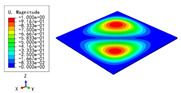	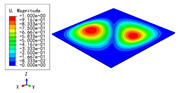	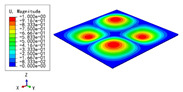
	1965.0 Hz	3553.4 Hz	3769.1 Hz	5062.7 Hz
2D-EPM	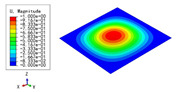	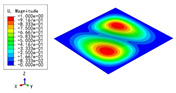	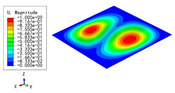	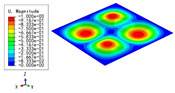
	1977.0 Hz (0.61%)	3534.4 Hz (0.53%)	3712.3 Hz (1.51%)	4919.2 Hz (2.83%)

**Table 14:** Comparison of the first four free vibrations of the orthogrid HSP under CCCC boundary conditions predicted by different models.

Model	1	2	3	4
3D-FEM	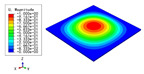	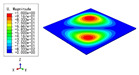	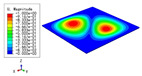	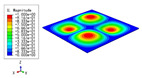
	2273.3 Hz	4187.4 Hz	4374.0 Hz	6248.4 Hz
2D-EPM	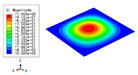	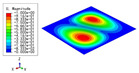	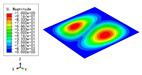	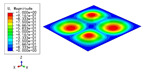
	2364.9 Hz (4.03%)	4324.1 Hz (3.26%)	4324.1 Hz (1.14%)	5839.1 Hz (6.55%)

**Table 15:** Comparison of the first four free vibrations of the X-shaped HSP under CCCC boundary conditions predicted by different models.

Model	1	2	3	4
3D-FEM	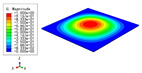	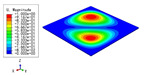	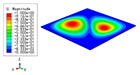	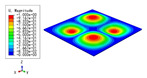
	2059.3 Hz	3746.3 Hz	3751.6 Hz	5134.6 Hz
2D-EPM	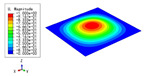	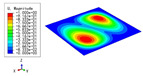	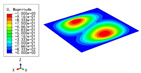	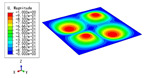
	2065.2 Hz (0.29%)	3745.1 Hz (0.03%)	3745.1 Hz (3.26%)	5073.4 Hz (1.19%)

**Table 16:** Comparison of the first four free vibrations of the resized triangular HSP under CCCC boundary conditions predicted by different models.

Model	1	2	3	4
3D-FEM	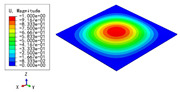	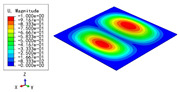	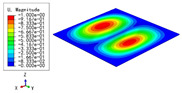	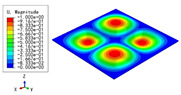
	2075.9 Hz	3657.5 Hz	3920.1 Hz	5190.9 Hz
2D-EPM	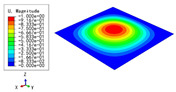	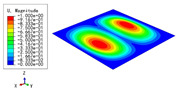	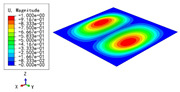	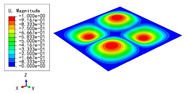
	2107.4 Hz (1.52%)	3731.9 Hz (2.03%)	3933.9 Hz (0.35%)	5181.9 Hz (0.02%)

## Data Availability

Data available on request due to restrictions, e.g., privacy or ethical. The data presented in this study are available on request from the corresponding author. The data are not publicly available due to subsequent analyzes and publications.
